# Advanced neuroimaging assessment of neurodegenerative dementia syndromes: A framework for comprehensive multimodal FDG-PET, MR-perfusion, and MR-diffusion analysis

**DOI:** 10.1016/j.nicl.2026.103964

**Published:** 2026-02-10

**Authors:** Joachim Strobel, Jan Kassubek, Wolfgang Thaiss, Sarah Straub-Anderl, Zeljko Uzelac, Sarah Jesse, Laura Michelberger, Christoph Solbach, Ambros J. Beer, Meinrad Beer, Georg Grön, Hans-Peter Müller, Nico Sollmann

**Affiliations:** a Department of Nuclear Medicine, University Hospital Ulm, Albert-Einstein-Allee 23, 89081 Ulm, Germany; b Department of Diagnostic and Interventional Radiology, University Hospital Ulm, Albert-Einstein-Allee 23, 89081 Ulm, Germany; c Department of Neurology, University Hospital Ulm, Oberer Eselsberg 45, 89081 Ulm, Germany; d Department of Psychiatry and Psychotherapy III, University Hospital Ulm, Leimgrubenweg 12-14, 89075 Ulm, Germany; e Department of Diagnostic and Interventional Neuroradiology, School of Medicine and Health, TUM Klinikum Rechts der Isar, Technical University of Munich, Ismaninger Str. 22, 81675 Munich, Germany; f TUM-Neuroimaging Center, TUM Klinikum Rechts der Isar, Technical University of Munich, Ismaninger Str. 22, 81675 Munich, Germany

**Keywords:** Positron emission tomography, Magnetic resonance imaging, Neurodegenerative diseases, Arterial spin labeling, Diffusion tensor imaging, Support vector machine

## Abstract

**Background:**

The reference for imaging in neurodegenerative dementia syndromes (NDS) is [^18^F]fluorodeoxyglucose ([^18^F]FDG) positron emission tomography (PET). Advanced multimodal magnetic resonance imaging (MRI) may complement PET-derived measures for differential diagnostics.

**Objectives:**

The aim of the present study was to evaluate a new methodological approach integrating metabolic, perfusion, and microstructural parameters from simultaneous FDG-PET/MRI to reveal different imaging signatures for different NDS subtypes.

**Materials and Methods:**

66 patients with NDS (Alzheimer’s disease: 28; behavioral frontotemporal dementia: 10; semantic variant primary progressive aphasia (PPA): 8; non-fluent variant PPA: 11; logopenic variant PPA: 9) and 10 subjects with subjective cognitive deficits (SCD) underwent combined FDG-PET/MRI with pseudo-continuous arterial spin labeling (pCASL) and diffusion tensor imaging (DTI). Standardized uptake values (SUV), cerebral blood flow (CBF), and fractional anisotropy (FA) were used to separate a respective NDS subgroup from all other NDS subgroups and from SCD based on a support vector machine (SVM) applied on region of interest (ROI)-based parameters.

**Results:**

White matter alterations directly adjacent to the regions of alterations in SUV and CBF were identified. The SVM analysis on ROI-based parameters reached accuracies between 81% and 94% for separating an NDS subgroup from all other NDS subgroups and from SCD.

**Conclusions:**

Multimodal neuroimaging by combination of FDG-PET and MRI shows potential to further advance the diagnostic spectrum in NDS. Since particularly early diagnosis of NDS remains key for effective treatment and management of patients with NDS, the present framework appears promising to be developed further until it aligns and integrates with clinical routine.

## Highlights

• New approach integrating metabolic, perfusion, and microstructural imaging in NDS.
• FA was altered in WM adjacent to regions of GM alterations (rSUV, rCBF)
• Multimodal SVM reached 81% to 94% accuracy for separating NDS from SCD.
• Advanced MRI may complement PET for differential diagnoses in future.

## 1 Introduction

Acquisition of multimodal imaging by combined positron emission tomography and magnetic resonance imaging (PET/MRI) represents a pivotal advancement in medical imaging, merging the metabolic insights from PET with the anatomical precision and functional assessment by MRI. This hybrid modality is particularly impactful in the realm of neurodegenerative dementia syndromes (NDS) such as Alzheimer's disease (AD) and frontotemporal dementia (FTD) with its subtypes of primary progressive aphasia (PPA) and its behavioral variant (bvFTD) (Scheltens et al., 2021, Gorno-Tempini et al., 2011, Rascovsky et al., 2011). These diseases are characterized by structural, functional, and/or metabolic changes, which can manifest in certain spatial patterns of affected brain areas that may be characteristic neural signatures of the corresponding NDS (Muddapu et al., 2020).

In NDS, [^18^F]fluorodeoxyglucose ([^18^F]FDG)-PET is widely used for assessing glucose metabolism, serving as a surrogate marker of neuronal integrity, with the spatial pattern of metabolic changes helping in diagnosing and discriminating between NDS (Minoshima et al., 2022). Previous work has shown certain spatial patterns for corresponding NDS diagnoses, such as for example hypometabolism of the parietal lobe, temporal lobe (including the hippocampus), and of some aspects of the frontal lobe in patients with AD, which represents the most frequent form of NDS (Na et al., 2024). Furthermore, recent ^18^F-THK5351 PET scanning of tau protein deposits showed affection of bilateral medial and lateral temporal and inferior parietal regions, dorsolateral prefrontal cortices, and the precuneus as a characteristic pattern for AD or PPA (Scheltens et al., 2021, Na et al., 2024, Routier et al., 2018). Other NDS tend to show different imaging patterns in glucose metabolism, such as bvFTD with bilateral frontal and prefrontal reductions, or left-predominant alterations in the anterior temporal cortices in semantic variant PPA (svPPA) (Cleland et al., 2021, Minoshima et al., 2022). Alterations in the left temporal-parietal junction and left inferior, middle, and superior temporal regions appear indicative of the logopenic variant PPA (lvPPA), while affection of the left inferior frontal gyrus, middle frontal gyrus, and supplementary motor area appears characteristic for the non-fluent variant PPA (nfvPPA) (Minoshima et al., 2022, Cleland et al., 2021).

PET is considered the imaging reference standard for assessing NDS. However, it cannot cover concurrent alterations in other domains of putative diagnostic relevance, such as perfusion or microstructure. These alterations can be captured by advanced MRI techniques such as arterial spin labeling (ASL) and diffusion tensor imaging (DTI), respectively (Sollmann et al., 2024, Müller and Kassubek, 2024, Chen et al., 2024, Bi et al., 2024, Li et al., 2022, Ceccarini et al., 2020, Zimny et al., 2015). Specifically, MRI with ASL can assess cerebral blood flow (CBF) by utilizing arterial blood-water as an endogenous tracer, thus providing information about perfusion of predominantly gray matter (GM) (Hoffmann et al., 2025, Sollmann et al., 2024, Lindner et al., 2023, Grade et al., 2015, Alsop et al., 2015). Previous ASL work in NDS has shown reductions in both global and regional CBF for patients with AD (Alsop et al., 2000). Furthermore, a consistent pattern of reduced CBF has been registered in key regions implicated in AD, including the cingulate cortex, precuneus, parietal lobes, inferior frontal regions, and the hippocampus (Meng et al., 2023, Zhang et al., 2017). Regarding white matter (WM), axonal architecture underlies structural connectivity in the brain, facilitating communication especially between cortical regions (Müller et al., 2007, Sampaio-Baptista and Johansen-Berg, 2017). In this regard, DTI permits *in-vivo* assessment of WM microstructure, revealing structural abnormalities such as axonal damage or demyelination (Müller et al., 2007, Le Bihan et al., 1986), representing relevant mechanisms in NDS (Müller et al., 2007, Le Bihan et al., 1986).

Previous studies have already indicated that multimodal integration may outperform PET only imaging in certain scenarios, demonstrating superior specificity and greater predictive value for NDS (Okazawa et al., 2024, Schwarz, 2021). A multimodal approach with combined PET/MRI, however, may not only help in differentiating between NDS by providing additional information about perfusion abnormalities or WM microstructure, but may also further improve diagnostic accuracy, particularly in the context of an as-early-as-possible diagnosis (Chen et al., 2024, Bi et al., 2024, Lee et al., 2024, Bergamino et al., 2024, Chen et al., 2023, Torso et al., 2021, Torso et al., 2020, Maggipinto et al., 2017, Mandelli et al., 2014). While previous studies have explored various combinations of PET with structural MRI or ASL (Dolui et al., 2020, Jack et al., 2018, Sun et al., 2022), no research to date has systematically integrated FDG-PET, ASL, and DTI within a unified PET/MRI-based framework.

Against this background, the aim of the current study was to demonstrate a first step into this direction by presenting a comprehensive methodological framework, which simultaneously reveals specific patterns in glucose metabolism, CBF, and WM microstructure in different NDS subtypes by combining PET, ASL, and DTI simultaneously acquired on a PET/MRI device.

## 2 Materials and methods

### 2.1 Data workflow

**Fig. 1:**
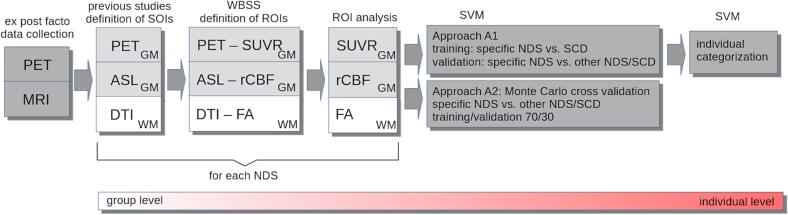
Schematic workflow. Ex post facto positron emission tomography/magnetic resonance imaging (PET/MRI) data collection according to defined protocols. The structures of interest (SOIs) were defined according to previous studies at the group level in various neurodegenerative dementia syndromes (NDS). The definition of regions of interest (ROIs) was based on SOIs and multimodal whole brain-based spatial statistics (WBSS) analysis (standardized uptake value ratio [SUVR] from PET / relative cerebral blood flow [rCBF] from arterial spin labeling [ASL] / fractional anisotropy [FA] from diffusion tensor imaging [DTI]) of the current study. After parameterization of individual multimodal data by ROI analysis, subject categorization was performed by a support vector machine (SVM) using two approaches (A1 and A2): A1 used the subjective cognitive decline (SCD) group for training the SVM for comparison with a specific NDS and performed validation of the SVM by comparison of a specific NDS with other NDS and SCD; A2 used a Monte Carlo cross validation in a training/validation ratio of 70:30 comparing a specific NDS with other NDS and SCD. In a last step, a suggestion for individual categorization was provided.

A schematic workflow is presented in Fig. 1. The data were collected *ex post facto* (section 2.2.) according to defined protocols (section 2.3.). Structures of interest (SOIs) were defined according to previous group-level studies in various NDS (section 2.5.). The SOIs were confirmed by whole brain-based spatial statistics (WBSS) at the group level (section 2.6.), and specific regions of interest (ROIs) were delineated (section 2.7.). Finally, a support vector machine (SVM) (2.8, 2.9) was applied for categorization of results at the individual level.

### 2.2 Study population

This *ex post facto* monocentric study was approved by the local institutional review board (registration number 164/24), certifying that the study complies with the Declaration of Helsinki and its amendments or equivalent ethical standards. The requirement for written informed consent was waived due to the retrospective study design.

Sixty-six consecutive patients with NDS (AD, bvFTD, and variants of PPA) and ten subjects with subjective cognitive deficits (SCD) were selected from the local database (picture archiving and communication system − PACS), with the requirement that all participants have been examined with the identical PET/MRI acquisition protocol on the same 3-T PET/MRI system (acquisition time interval: December 2022 to May 2024). All patients have been referred for PET/MRI based on clinical indications and because of recent cognitive decline and suspicion of NDS. All NDS cases were clinically diagnosed according to standardized criteria: AD related to the diagnostic criteria of the Alzheimer's Association Workgroup, and clinical diagnoses of PPA and bvFTD were related to guidelines reported by Gorno-Tempini and coworkers (Gorno-Tempini et al., 2011) and Rascovsky and coworkers (Rascovsky et al., 2011).

Selection of study participants was guided by excluding cases with indications of severe movement artifacts, incomplete imaging data, and gross structural brain abnormalities (such as intracranial tumors). Patients underwent routine clinical, neurological, and neuropsychological examinations during the clinical workup. Fifty patients (20 AD, 7 bvFTD, 8 svPPA, 8 nfvPPA, 7 lvPPA) and five subjects with SCD completed the Mini-Mental State Examination (MMSE) (Arevalo-Rodriguez et al., 2021, Creavin et al., 2016, Mitchell, 2017). Subjects with SCD but without any clinical diagnosis of NDS served as an analogue of a control group.

The study cohort included the following diagnostic categories: 10 subjects with SCD (5f / 52 ± 16 years), 28 patients with AD (11f / 66 ± 7 years), 10 patients with bvFTD (4f / 62 ± 10 years), 8 patients with svPPA (3f / 71 ± 7 years), 11 patients with nfvPPA (4f / 70 ± 8 years), and 9 patients with lvPPA (4f / 65 ± 5 years). Characteristics of the study sample are summarized in Table 1.

**Table 1:** Subject characteristics.

	**SCD**	**AD**	**bvFTD**	**svPPA**	**lvPPA**	**nfvPPA**	**p**
**age / years**	52.2 ± 15.8	65.7 ± 7.3	61.5 ± 10.3	71.1 ± 6.7	65.3 ± 5.4	69.6 ± 7.6	0.01
**sex (m/f)**	5 / 5	17 / 11	6 / 4	5 / 3	5 / 4	7 / 4	−
**MMSE**	28.2 ± 1.6	23.5 ± 3.7	20.3 ± 9.8	23.5 ± 5.5	18.4 ± 9.3	28.4 ± 0.9	0.02
**disease duration / years**	−	3.2 ± 2.8	2.6 ± 1.8	7.0 ± 4.1	2.0 ± 1.1	3.7 ± 1.6	0.02

Values are given in mean ± standard deviation. p-values were calculated by Kruskal-Wallis test. Abbreviations: subjective cognitive deficits (SCD), Alzheimer’s disease (AD), behavioral frontotemporal dementia (bvFTD), semantic variant primary progressive aphasia (svPPA), logopenic variant PPA (lvPPA), non-fluent variant PPA (nfvPPA), mini-mental state examination (MMSE).

### 2.3 Image acquisitions

The PET and MRI sequences were acquired during a ∼45-min scan of dynamic PET acquisition in list mode on an integrated 3-T PET/MRI scanner (Biograph mMR, Siemens Healthineers, Erlangen, Germany) using a 12-channel head coil. All subjects underwent the identical MRI acquisition protocol with PET acquisitions and including a three-dimensional (3D) T1-weighted magnetization-prepared rapid gradient echo (MPRage), diffusion-weighted imaging (DWI), and pseudo-continuous ASL (pCASL) sequence.

The 3D high-resolution MPRage sequence was acquired as an anatomical reference with the following parameters: repetition time (TR)/echo time (TE) = 1900 ms/3.29 ms, inversion time = 900 ms, flip angle = 9°, voxel size = 0.5 × 0.5 × 0.9 mm^3^, and 192 slices in sagittal orientation.

#### 2.3.1 PET acquisition

Patients were placed in a darkened, soundproof room one hour before the PET/MRI examination. Weight-adapted doses were used (3.0 MBq/kg; range of approximately 200 MBq to 500 MBq, with a half-life of approximately 110 min), and the radiopharmaceutical was applied after 30 min, thus allowing for additional 30 min of uptake before the examination was conducted.

List-mode PET data were acquired (236 ± 50 MBq), and were reconstructed from minute 35 to minute 45 to one image volume (344 × 344 × 127 matrix) using Siemens SyngoVia (VB60S; Siemens Healthineers, Erlangen, Germany), HF01 tools, and an iterative algorithm (ordered subset expectation maximization with point-spread function model, 3 iterations, 21 subsets, 3D Gaussian filter with a full-width-half-maximum [FWHM] of 2 mm, and zoom factor of 2.5). Scatter, decay, and dead-time corrections were applied to all PET data. Attenuation correction was performed using a magnetic resonance-based attenuation correction (MRAC) high-resolution approach for the brain, employing a Dixon sequence (TE = 1.28/2.51 ms). The reconstructed PET images had a spatial resolution of 1.04 × 1.04 × 2.03 mm^3^ (x/y/z). The temporal resolution of ten minutes was chosen as this interval temporally aligned with on- and offset of the MRI-based perfusion sequence.

#### 2.3.2 ASL acquisition

A pCASL sequence with a single-shot 3D gradient and spin echo (GRASE) readout was acquired starting around minute 35 (parallel to the FDG-PET acquisition) using the following parameters: TR = 4110 ms, TE = 37.9 ms, permuted post-labeling delays (PLDs) of 1.5 s / 1.8 s / 2.0 s, and acquisition of 36 label and control pairs (i.e., 12 label/control pairs per each PLD). The number of slices was 40 with 10% oversampling, acquisition of 64 label and control pairs, 40 slices with 10% oversampling, no Partial Fourier in the slice-encoding direction, parallel imaging acceleration factor of 2 along the phase-encoding direction with generalized autocalibrating partial parallel acquisition (GRAPPA) reconstruction, refocusing flip angle of 120 degrees, bandwidth = 2170 Hz/pixel, and 3.9 × 3.9 × 3.6 mm^3^ voxel size. The labeling consisted of a 1.5 s train of radiofrequency (RF) pulses with gaps (pseudo-continuous), using two non-selective inversion pulses for suppression of static background signal. The labeling plane was positioned orthogonally to the course of the internal carotid arteries (ICAs) and approximately 9 cm beneath the first imaging plane. For CBF quantification, proton-density images (M_0_) using the pCASL sequence minus label and background suppression and RF pulses with an additional 2000 ms of TR were acquired. The total acquisition time was 10.33 min.

#### 2.3.3 DWI acquisition

Whole-brain DWI was performed axially, using a two-dimensional (2D) single-shot echo planar imaging (EPI) sequence with 64 gradient directions (b = 1000 s/mm^2^ and b = 0 s/mm^2^). Imaging parameters included a 2D matrix of 108 x 108, with 86 contiguous slices and a voxel resolution of 2.1 x 2.1 x 1.8 mm^3^, TE = 94 ms, and TR = 13,500 ms. An oversampling factor of 100% was applied, with 6/8 Partial Fourier acquisition in the slice-encoding direction. Parallel imaging was employed with an acceleration factor of 2 along the phase-encoding direction (GRAPPA). The bandwidth was set at 1446 Hz/pixel, and the total scan duration was 15.10 min.

### 2.4 Pre-processing

The pre- and post-processing steps of the analysis were performed using the Tensor Imaging and Fiber Tracking (TIFT) software (Müller et al., 2007). Non-linear spatial normalization was performed using study-specific templates, oriented to the Montreal Neurological Institute (MNI) stereotaxic standard space by the use of the “Colin brain template” (Brett et al., 2002, Holmes et al., 1998), and preserving directional information in case of DTI data processing (Müller et al., 2007). This normalization procedure has been well established for DTI in previous large-scale work (Müller et al., 2016), and it has been adapted to the normalization of ASL data as well as to PET data, each with modality- and study-specific templates.

Standardized uptake values (SUV) were initially derived from the PET data by normalization to injected dose and patient body weight. Subsequently, SUV ratio (SUVR) maps were computed by further normalizing regional SUV values to the mean whole-brain uptake, which served as the reference. All subsequent analyses were based on SUVR values. The resulting maps were used for spatial smoothing with an isotropic Gaussian kernel (8 mm FWHM) (Unrath et al., 2010), which was used to obtain a balance between sensitivity and specificity and to accommodate individual anatomical variability.

After motion correction, maps of CBF were generated from the pCASL data (Alsop et al., 2015, Buxton et al., 1998). Each voxel’s CBF values were normalized to average CBF across a whole-brain mask to standardize intensity (Tosun et al., 2016a, Tosun et al., 2016b, Wang et al., 2005). Data were then interpolated onto a 1-mm isotropic voxel grid to enhance spatial resolution for subsequent analyses, and relative CBF (rCBF) maps were computed accordingly. Given that images were obtained at three PLDs, rCBF maps from the various PLDs were averaged for a more robust representation of perfusion across varying PLDs. The normalized rCBF maps were smoothed using an isotropic Gaussian filter (8 mm FWHM) to match the intrinsic smoothness of the data (Tosun et al., 2016b).

For DWI data, an established quality control protocol was followed to compensate for corrupted gradient directions as well as motion artifacts prior to correction of eddy current-induced geometric distortions (Müller et al., 2014). Data were interpolated onto a 1-mm isotropic voxel grid for all further analyses (Müller et al., 2016). For DTI analysis, fractional anisotropy (FA) maps were calculated from MNI-normalized DTI data, and a Gaussian smoothing filter of 8 mm FWHM was applied to the normalized individual FA maps to balance sensitivity and specificity (Unrath et al., 2010). For FA maps, age is a contributing factor in FA analyses (Behler et al., 2021). Thus, FA maps were corrected for age according to a standardized protocol (Müller et al., 2016).

### 2.5 Definition of structures of interest

The selection of SOIs was based on established areas of hypometabolism, hypoperfusion, or microstructural changes identified in the literature and relevant to the NDS under investigation: AD, bvFTD, svPPA, nfvPPA, and lvPPA.

#### 2.5.1 SOI definitions for AD

As reported in a recent meta-analysis of PET data, patients with AD showed hypometabolism in the parietal lobe, temporal lobe (including the hippocampus), and some parts of the frontal lobe (Na et al., 2024). In a recent PET study, AD patients showed greater affection of bilateral medial and lateral temporal and inferior parietal regions, dorsolateral prefrontal cortices, and the precuneus (Strobel et al., 2024, Nam et al., 2018). Moreover, ASL showed reduced perfusion in the temporal lobe even at the single-subject level (Du et al., 2006).

In AD patients, the cingulum, temporal lobe (including the hippocampal part of the cingulum), and splenium of the corpus callosum showed the most pronounced alterations in FA maps according to previous work (Nir et al., 2013). In a recent systematic review, AD patients showed extensive microstructural impairment, structural disconnection, and topological abnormalities mainly in the corpus callosum, fornix, and medial temporal lobes, including the hippocampus and cingulum (Chen et al., 2023).

#### 2.5.2 SOI definitions for bvFTD

Using PET, patients with bvFTD showed higher bilateral affection of the medial frontal, dorsolateral prefrontal, and orbitofrontal cortex, anterior cingulate, insula, anterior inferior temporal, and striatum regions at the group level (Nam et al., 2018). In a combined PET/ASL study, bvFTD patients showed predominantly frontotemporal hypoperfusion (Tosun et al., 2016b). Alterations of WM in bvFTD were predominantly observed in the frontal lobes (Kassubek, 2018, Rascovsky et al., 2011, Tosun et al., 2016b, Agosta et al., 2025).

#### 2.5.3 SOI definitions for PPA

A previous study reported bilateral but left-predominant hypometabolism in the anterior temporal cortices, extending to the left cingulate and toward middle/posterior temporal regions in svPPA patients (Routier et al., 2018). Furthermore, lvPPA patients showed alterations in the left temporal-parietal junction and left inferior, middle, and superior temporal regions extending toward anterior temporal cortices (Routier et al., 2018). By contrast, nfvPPA patients demonstrated hypometabolism in aspects of the left inferior frontal gyrus, middle frontal gyrus, and supplementary motor area (Routier et al., 2018). The same study showed the following tract-based microstructural alterations using DTI: svPPA patients showed bilateral alterations in tracts making connections with the anterior temporal cortex (i.e., bilateral uncinate fasciculus); lvPPA patients demonstrated alterations of the left superior longitudinal fasciculus and left inferior frontal-occipital fasciculus; nfvPPA patients demonstrated alterations of the left uncinate fasciculus, left anterior thalamic radiation, and the temporal part of the right superior longitudinal fasciculus (Routier et al., 2018).

### 2.6 Whole brain-based spatial statistics

To estimate whether the respective NDS sample of this study showed alterations of the different measurement modalities according to the SOI positions selected, a voxel-wise statistical comparison using WBSS was performed (Müller et al., 2016). This was done between each of the different NDS subgroups and the SCD group. Statistical results were corrected for multiple comparisons using a false-discovery rate (FDR) at a significance level of p < 0.05 (Genovese et al., 2002). Further reduction of the alpha error was performed by a spatial correlation algorithm that eliminated isolated voxels or small isolated groups of voxels in the size range of the smoothing kernel, leading to a threshold cluster size of 256 voxels (256 mm^3^) (Unrath et al., 2010).

### 2.7 Region of interest definitions and analyses

Although the numerous SOIs for each NDS entity would have permitted a corresponding greater number of ROIs, selections had to be performed to reduce the number of features for the intended categorization analyses using an SVM, particularly considering the lower sample sizes of the PPA subgroups. Furthermore, parameters derived from GM and WM should be in close vicinity.

The ROI analyses were performed by arithmetically averaging the relevant parameter (i.e., SUVR from PET, FA from DTI, rCBF from ASL) for each modality and each subject within a given spherical region, yielding mean ROI-related average values (i.e., <SUVR>_ROI_, <FA>_ROI_, <rCBF>_ROI_). The radius of the spherical ROIs was set at 30 mm by default (30 voxels in a 1-mm grid, except for one ROI which was set to 20 mm) to achieve a good balance between sensitivity and specificity. Especially for FA analysis, only voxels with an FA value greater than 0.2 were considered (Kunimatsu et al., 2004). For SUVR and rCBF, no thresholds were applied during analyses.

### 2.8 Support vector machine and Monte Carlo cross validation

Among techniques for multi-parametric group separation (including artificial intelligence [AI] methods), an SVM shows applicability especially for separation of smaller samples. Hence, an SVM approach with a scalar product kernel (Evgeniou and Pontil, 2001) was used to calculate a three- to five-dimensional hyperplane with one or two ROIs for each NDS for the three parameters <SUVR>_ROI_, <FA>_ROI_, <rCBF>_ROI_, depending on the corresponding NDS.

Due to lower sample sizes, and due to the main aim of the study demonstrating the feasibility of the present multimodal imaging framework, we deviated, in a first approach (A1), from the classical training/validation scheme (with permutations). Therefore, we adjusted the SVM for maximum specificity (targeting to reach 100%) in separating each NDS subgroup from the SCD group (that way acting as a training set). The resulting hyperplane was then used to separate a specific NDS subgroup of interest from all other NDS subgroups (that way acting as a validation set).

In a second approach (A2), Monte Carlo cross validation was used to separate a specific NDS from all other NDS and SCD, with 1000 SVM runs performed, dividing both groups (specific NDS vs other NDS and SCD) in a 70:30 ratio into training and validation.

Beside sensitivity and specificity, accuracy was calculated and defined as:

A=n/N

where *n* is the number of correctly classified patients of the corresponding NDS subgroup and *N* the total number of NDS subjects.

The SVM was performed for PET only (<SUVR>_ROI_), the combination of MRI modalities (<FA>_ROI_ and <rCBF>_ROI_), and the combination of all three modalities (<SUVR>_ROI_ and <FA>_ROI_ and <rCBF>_ROI_).

### 2.9 Subject categorization targeting support of diagnosis

To estimate how the herein used multimodal imaging approach may impact a patient categorization targeting to guide in the diagnosis, a categorization to the respective clinical NDS diagnosis was performed based on multimodal imaging parameters (using approach A1). If the categorization of a patient coincided with the initial clinical diagnosis in the SVM analysis, this patient was defined as “correctly categorized” (1=”TRUE” – Supplementary Table 1). The composite percentage was defined as

$$C=N_{\mathrm{TRUE}}/\left(N_{\mathrm{FALSE}}+N_{\mathrm{TRUE}}\right)$$

where *N_TRUE_* is the number of correctly categorized patients and *N_FALSE_ + N_TRUE_* is the total number of subjects.

### 2.10 Statistics and association analysis

For demographics and results from MMSE assessments, between-group differences were calculated using Kruskal-Wallis tests, with a threshold for statistical significance at p < 0.05. For between-group comparisons of the individual ROI-specific or averaged modality-specific values, Welch or Mann-Whitney U tests (in case of non-parametric data distributions) were performed.

Associations to MMSE scores were calculated with the values of the ROI analyses for the respective diagnostic category using Pearson correlation analyses, and statistical significance was inferred after correction for multiple comparisons using the Bonferroni-Holm method (Aickin and Gensler, 1996).

## 3 Results

### 3.1 Whole brain-based spatial statistics

**Fig. 2:**
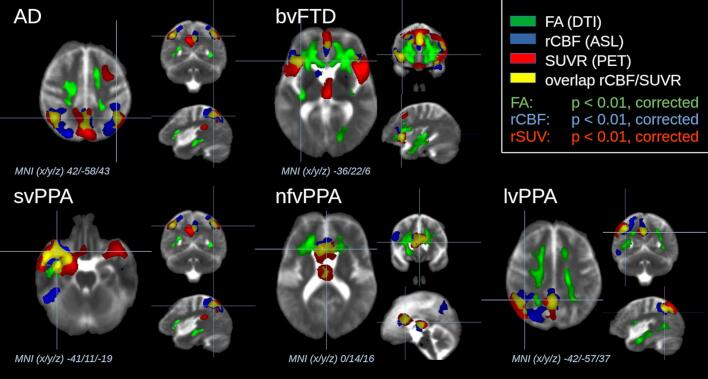
Voxel-wise statistical comparison by whole brain-based spatial statistics (WBSS). Positron emission tomography (PET)-derived standardized uptake value ratio (SUVR, red), arterial spin labeling (ASL)-derived relative cerebral blood flow (rCBF, blue), and diffusion tensor imaging (DTI)-derived fractional anisotropy (FA, green) of neurodegenerative dementia syndromes (NDS) were compared with subjects with subjective cognitive deficits (SCD). Exemplary representative axial, coronar, and sagittal slices are shown to demonstrate the close spatial relationships of gray matter (GM) alterations (i.e., SUVR, rCBF) and white matter (WM) alterations (i.e., FA). The spatial overlap of results of SUVR and rCBF is displayed in yellow. ASL and PET showed predominantly overlapping results in GM albeit with different cluster extensions for PET or ASL, depending on the respective NDS subgroup. DTI showed voxel clusters of alterations predominantly in the WM adjacent to the GM alteration clusters. Significance thresholds were adapted to the respective modality and the respective NDS (p < 0.01, corrected). Abbreviations: Alzheimer’s disease (AD), behavioral frontotemporal dementia (bvFTD), semantic variant primary progressive aphasia (svPPA), logopenic variant PPA (lvPPA), non-fluent variant PPA (nfvPPA), Montreal Neurological Institute (MNI) coordinate frame. (For interpretation of the references to colour in this figure legend, the reader is referred to the web version of this article.)

Voxel-wise statistical comparisons at the group level of SUVR, FA, and rCBF by WBSS were performed for each NDS subgroup against SCD (Fig. 2). For all NDS subgroups, high regional coincidences could be detected when using parameters SUVR and rCBF, albeit with different cluster extensions for PET and ASL, and also indicating left-lateralized dominance of findings for svPPA and lvPPA. Furthermore, for bvFTD, svPPA, and nfvPPA, FA was altered in WM adjacent to the regions of alterations in SUVR and rCBF, while for AD and lvPPA, FA showed alterations in WM regions where WM tracts are targeting regions of alterations in SUVR and rCBF, that way demonstrating spatial relationships of GM alterations (i.e., SUVR, rCBF) and WM alterations (i.e., FA).

### 3.2 Region of interest definitions

**Fig. 3:**
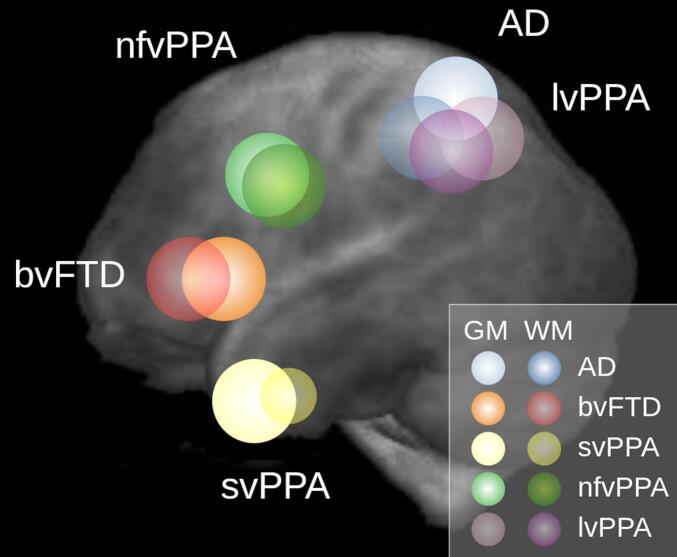
Schematic overview of the neurodegenerative dementia syndromes (NDS)-specific region of interest (ROI) localizations. The sagittal projectional view was used to illustrate the positions of the spherical ROIs. Abbreviations: gray matter (GM), white matter (WM), Alzheimer’s disease (AD), behavioral frontotemporal dementia (bvFTD), semantic variant primary progressive aphasia (svPPA), logopenic variant PPA (lvPPA), non-fluent variant PPA (nfvPPA).

Based on a combination of structures of interest (section 2.5.) and WBSS results (section 3.1.), ROIs were delineated (schematically displayed in Fig. 3, extensions and MNI coordinates are summarized in Table 2).

**Table 2:** Region of interest (ROI) localization and sizes for structures of interest (SOI) in Montreal Neurological Institute (MNI) coordinates.

	**MNI (x/y/z), R(mm)**	**MNI (x/y/z), R(mm)**	**Structure of Interest (SOI)**
**AD**			
GM (PET)	0/-70/36, 30		parietal lobe
GM (ASL)	40/-56/46, 30	−40/-56/46, 30	inferior parietal lobe
WM (DTI)	22/-43/37, 30	–22/-43/37, 30	inferior parietal lobe
**bvFTD**			
GM (PET)	0/32/35, 30		frontal lobe, medial frontal gyrus
GM (ASL)	41/19/-1, 30	−41/19/-1, 30	frontal lobe, inferior frontal gyrus
WM (DTI)	24/33/3, 30	−24/33/3, 30	frontal lobe, adjacent to inferior frontal gyrus
**svPPA**			
GM (PET)	41/11/-21, 30	−41/11/-21, 30	temporal lobe, superior temporal gyrus
GM (ASL)	−46/11/-19, 30		temporal lobe, superior temporal gyrus
WM (DTI)	40/-7/-19, 20	−40/-7/-19, 20	temporal lobe, adjacent to superior temporal gyrus
**nfvPPA**			
GM (PET, ASL)	−4/13/16, 30		adjacent to callosal area I
WM (DTI)	−25/-15/40, 30		frontal lobe, adjacent to cingulate gyrus
**lvPPA**			
GM (PET, ASL)	−40/-60/43, 30		inferior parietal lobe, BA 40
WM (DTI)	25/-55/32, 30		adjacent to BA 40

Abbreviations: Brodmann Area (BA), gray matter (GM), white matter (WM), ROI radius in mm (R), Alzheimer’s disease (AD), behavioral frontotemporal dementia (bvFTD), semantic variant primary progressive aphasia (svPPA), logopenic variant PPA (lvPPA), non-fluent variant PPA (nfvPPA), diffusion tensor imaging (DTI), arterial spin labeling (ASL), positron emission tomography (PET).

**Fig. 4:**
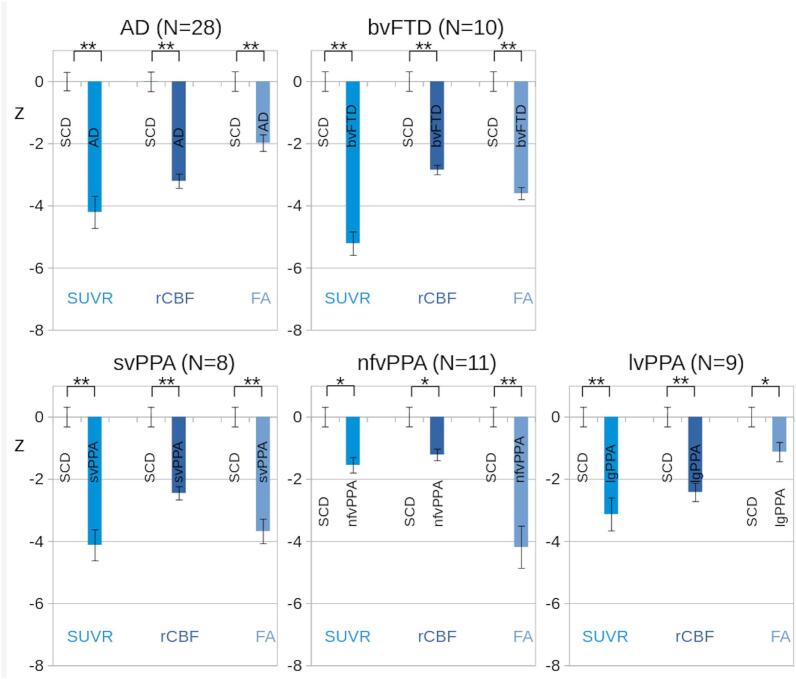
Comparisons of region of interest (ROI)-averaged values. ROI-averaged <SUVR>_ROI_, <rCBF>_ROI_, and <FA>_ROI_ values of the neurodegenerative dementia syndromes (NDS; AD, bvFTD, svPPA, nfvPPA, and lvPPA) were compared with subjects with subjective cognitive deficits (SCD). For illustration purposes (comparability across groups and modalities), results were scaled to the average of SCD (accordingly, the average of SCD was 0 and standard error was 1). Average values of patients with NDS after scaling are represented by blue bars, and error bars show the standard error of the mean. *p < 0.01, corrected; **p < 0.001, corrected. Abbreviations: Alzheimer’s disease (AD), behavioral frontotemporal dementia (bvFTD), semantic variant primary progressive aphasia (svPPA), logopenic variant PPA (lvPPA), non-fluent variant PPA (nfvPPA). (For interpretation of the references to colour in this figure legend, the reader is referred to the web version of this article.)

Fig. 4 shows comparisons of modality-specific aggregates between the NDS subgroups (AD, bvFTD, svPPA, nfvPPA, and lvPPA) according to ROI localizations. There were statistically significant differences in PET-derived <SUVR>_ROI_, ASL-derived <rCBF>_ROI_, and DTI-derived <FA>_ROI_ data between SCD and each of the five NDS subgroups, respectively. Hence, regarding the specific ROIs considered for each NDS, parameters representative of metabolism, perfusion, as well as WM microstructure were significantly different between SCD and NDS patients.

### 3.3 Support vector machine approach of modality-specific parameters

For approach A1, the adjustment of the SVM for maximum specificity (targeting to reach 100%) was possible in 20 out of the 25 separations of an NDS subgroup from the SCD group. Four separations reached 90%, and one separation reached only 70% when keeping sensitivity above a reasonable threshold of 50%. That way, the SVM was reduced to a hyperplane that separated the (almost) whole SCD group from the NDS subgroup. This hyperplane was then used to separate the NDS subgroup of interest from all other subgroups. The SVM (or simply hyperplane separation) results from ROI analyses of modality-specific parameters are summarized in Table 3.

**Table 3:** Results of the support vector machine (SVM) approach for classification of neurodegenerative dementia syndromes (NDS). Upper panel (approach A1): a) PET/MRI(ASL/DTI) results after categorization by an SVM; average accuracy for comparison with SCD was 91.4 and for comparison vs all other pathologies was 78.2. b) PET results after categorization by an SVM; average accuracy for comparison with SCD was 92.0 and for comparison with all other pathologies was 78.1. c) MRI(ASL/DTI) results after categorization by an SVM; average accuracy for comparison with SCD was 87.1 and for comparison with all other pathologies was 70.8. Lower panel (approach A2): d) PET/MRI(ASL/DTI) results after categorization by an SVM; average accuracy for comparison of specific NDS with other NDS and SCD was 76.7. e) PET results after categorization by an SVM; average accuracy for comparison of specific NDS with other NDS and SCD was 80.1. f) MRI(ASL/DTI) results after categorization by an SVM; average accuracy for comparison of specific NDS with other NDS and SCD was 69.0.

	**sens./%**	**spec./%**	**acc./%**	**sens./%**	**spec./%**	**acc./%**	**sens./%**	**spec./%**	**acc./%**
**A1**	**a) PET/MRI(ASL/DTI)**			**b) PET**			**c) MRI(ASL/DTI)**		
**AD (N = 28)**									
vs. SCD	82.1	100.0	86.8	85.7	100.0	89.5	67.9	100.0	76.3
vs. all	82.1	79.2	80.3	85.7	83.3	84.2	67.9	62.5	64.5
**bvFTD (N = 10)**									
vs. SCD	100.0	100.0	100.0	100.0	100.0	100.0	90.0	100.0	95.0
vs. all	100.0	80.3	82.9	100.0	80.3	82.9	90.0	78.8	80.3
**svPPA (N = 8)**									
vs. SCD	87.5	100.0	94.4	100.0	100.0	100.0	75.0	100.0	88.9
vs. all	87.5	91.2	90.8	100.0	89.7	90.8	75.0	86.8	85.5
**nfvPPA (N = 11)**									
vs. SCD	63.6	100.0	81.0	72.7	90.0	81.0	63.6	100.0	81.0
vs. all	63.6	61.5	61.8	72.7	50.8	53.9	63.6	64.6	64.5
**lvPPA (N = 9)**									
vs. SCD	88.9	100.0	94.7	77.8	100.0	89.5	88.9	100.0	94.7
vs. all	88.9	73.2	75.0	77.8	79.1	78.9	88.9	55.2	59.2


**A2**	**d) PET/MRI(ASL/DTI)**			**e) PET**			**f) MRI(ASL/DTI)**		

**AD (N = 28)**									
training	77.1 ± 6.5	74.4 ± 4.8	75.4 ± 2.6	85.8 ± 3.6	83.2 ± 2.8	84.2 ± 2.2	62.0 ± 6.5	72.4 ± 4.9	68.6 ± 3.0
validation	74.8 ± 17.7	73.8 ± 14.0	74.2 ± 10.5	85.4 ± 13.2	83.8 ± 10.7	84.4 ± 8.3	60.5 ± 18.5	71.8 ± 13.4	67.5 ± 10.7
summary	76.6 ± 5.6	74.3 ± 4.3	75.1 ± 1.9	85.7 ± 0.5	83.3 ± 0.3	84.2 ± 0.2	61.6 ± 4.7	72.3 ± 3.5	68.3 ± 1.5
**bvFTD (N = 10)**									
training	99.2 ± 3.3	80.3 ± 2.7	82.6 ± 2.4	79.2 ± 8.7	94.0 ± 1.9	92.4 ± 1.9	85.9 ± 10.2	77.2 ± 3.8	78.2 ± 3.3
validation	98.7 ± 6.7	79.2 ± 9.6	82.7 ± 7.9	80.1 ± 20.1	94.2 ± 5.7	91.7 ± 5.8	87.2 ± 17.1	76.3 ± 10.1	78.2 ± 3.3
summary	99.1 ± 3.2	80.1 ± 1.6	82.6 ± 1.3	80.0 ± 0.3	94.1 ± 0.9	92.2 ± 0.8	86.3 ± 6.0	77.0 ± 2.5	78.2 ± 1.9
**svPPA (N = 8)**									
training	86.8 ± 7.3	97.0 ± 1.9	96.0 ± 1.8	87.3 ± 7.1	97.2 ± 1.3	96.2 ± 1.3	73.8 ± 13.1	84.3 ± 6.9	83.3 ± 5.9
validation	88.3 ± 21.3	95.6 ± 4.8	94.7 ± 5.0	88.7 ± 20.9	97.0 ± 4.1	96.0 ± 4.3	73.8 ± 30.5	82.9 ± 10.1	81.7 ± 9.0
summary	87.2 ± 2.0	96.7 ± 1.5	95.7 ± 1.2	87.6 ± 1.2	97.1 ± 0.9	96.1 ± 0.7	73.8 ± 9.6	84.0 ± 6.2	82.9 ± 4.8
**nfvPPA (N = 11)**									
training	63.6 ± 10.5	61.7 ± 8.5	62.0 ± 7.0	49.6 ± 13.3	53.2 ± 8.2	52.7 ± 6.0	61.3 ± 10.3	63.4 ± 8.6	63.1 ± 7.5
validation	58.7 ± 25.9	53.5 ± 13.2	54.4 ± 11.5	44.9 ± 27.5	44.4 ± 11.9	44.5 ± 9.6	56.9 ± 26.0	59.1 ± 11.7	58.7 ± 10.4
summary	57.1 ± 17.9	55.1 ± 18.2	55.4 ± 17.7	48.3 ± 9.5	51.3 ± 7.7	50.9 ± 5.2	55.2 ± 17.1	57.4 ± 18.5	57.1 ± 18.0
**lvPPA (N = 9)**									
training	78.3 ± 16.1	74.6 ± 5.6	75.0 ± 4.2	72.6 ± 11.6	77.4 ± 4.4	76.8 ± 4.2	71.2 ± 13.1	59.3 ± 9.0	60.7 ± 7.3
validation	76.3 ± 29.6	73.5 ± 10.9	73.9 ± 9.5	73.8 ± 30.5	78.5 ± 9.8	78.0 ± 8.9	67.8 ± 31.7	55.1 ± 12.4	56.7 ± 11.5
summary	77.9 ± 14.1	74.4 ± 5.0	74.8 ± 3.5	72.9 ± 7.0	77.6 ± 3.3	77.1 ± 2.8	69.2 ± 14.2	57.3 ± 11.1	58.7 ± 10.2
average NDS	79.6 ± 15.5	76.1 ± 14.9	76.7 ± 14.7	74.9 ± 15.9	80.7 ± 18.2	80.1 ± 17.9	69.2 ± 11.9	69.6 ± 11.9	69.0 ± 11.5

Abbreviations: sensitivity (sens.), specificity (spec.), accuracy (acc.), Alzheimer’s disease (AD), behavioral frontotemporal dementia (bvFTD), semantic variant primary progressive aphasia (svPPA), logopenic variant PPA (lvPPA), non-fluent variant PPA (nfvPPA), diffusion tensor imaging (DTI), arterial spin labeling (ASL), positron emission tomography (PET), magnetic resonance imaging (MRI).

In order to overcome problems with subgroup sizes as well as problems of using SCD instead of healthy controls, a training/validation analysis with Monte Carlo cross validation (approach A2) has been performed.

#### 3.3.1 SVM with PET only

For approach A1, the accuracy of PET only to distinguish between a respective NDS (AD, bvFTD, svPPA, nfvPPA, and lvPPA) and SCD was 92.0% on average, while it was 78.1% on average when distinguishing a specific NDS sample from the remaining NDS subgroups (Table 3). For approach A2, the accuracy of PET only to distinguish between a respective NDS and other NDS and SCD was 80.1% on average; an exception was accuracy in categorising nfvPPA where PET only showed accuracies around 50%, which could be judged as “separation failed” (Table 3).

#### 3.3.2 SVM with MRI (combined ASL/DTI data)

For approach A1, when comparing combined imaging by MRI (ASL/DTI) with PET only, ASL/DTI showed similar accuracy of 81% for nfvPPA comparison with SCD, and of 64.5% (PET only: 53.9%) for comparison with the other NDS subgroups. For lvPPA, accuracy was 94.7% (PET only: 89.5%) for comparison with SCD, and 59.2% (PET only: 78.9%) for comparison against the other NDS subgroups. For bvFTD vs SCD, the accuracy of 95% for ASL/DTI was similar to PET results (PET only: 100%), and of 80.3% (PET only: 82.9%) for comparison against the other NDS subgroups. For AD vs SCD, accuracy of 76.3% was inferior to PET only (89.5%) for comparison with SCD, and of 64.5% (PET only: 84.2%) for comparison against the other NDS subgroups. For svPPA vs SCD, accuracy of 88.9% was inferior to PET only (100.0%) for comparison with SCD, and of 85.5% (PET only: 90.8%) for comparison against the other NDS subgroups (Table 3).

For approach A2, accuracies derived from MRI were on average lower than accuracy for PET only, except for nfvPPA where PET only showed accuracies around 50%, which was judged as “separation failed” (Table 3).

#### 3.3.3 SVM with combined PET/ASL/DTI data

For approach A1, when comparing multimodal imaging (PET/ASL/DTI) with accuracies obtained from PET only, multimodal imaging showed similar accuracies (Table 3).

For approach A2, accuracies categorizing AD and bvFTD with combined PET/MRI (ASL/DTI) were on average lower than accuracy for PET only; for svPPA and lvPPA, accuracies for combined PET/MRI(ASL/DTI) were similar compared with accuracies for PET only except for nfvPPA where PET only showed “separation failed” (Table 3).

### 3.4 Estimation of the impact of multimodal imaging on patient categorization

According to the classification scheme of section 2.9**.**, in summary 95% of the participants were classified as “correctly categorized”. A summary of individual classification results is provided in Supplementary Table 1. This composite percentage is only a descriptive summary rather than a standalone accuracy metric.

### 3.5 Further descriptive statistics

Regarding associations of modality-specific ROI-averaged values for the NDS subgroups (AD, bvFTD, svPPA, nfvPPA, and lvPPA), moderate to strong significant associations were observed in AD between MMSE scores and <SUVR>_ROI_, <rCBF>_ROI_, as well as <FA>_ROI_, respectively (correlation coefficient range: 0.49 – 0.69, highest for ASL, p < 0.05; Supplementary Fig. 1). Furthermore, significant associations were observed in lvPPA between MMSE scores and <SUVR>_ROI_, <rCBF>_ROI_, as well as <FA>_ROI_, respectively (correlation coefficient range: 0.68 – 0.76, highest for PET, p < 0.05; Supplementary Fig. 1).

Supplementary Fig. 2 provides receiver operating characteristics (ROC) and area under the curve (AUC) for approach A1 for specific NDS vs other NDS and SCD.

## 4 Discussion

### 4.1 Multimodal imaging: parameterization in neurodegeneration

This study employed advanced multimodal neuroimaging techniques (i.e., FDG-PET, ASL, and DTI) to systematically investigate the neural signatures and regional pathological interplay between metabolic, perfusion, and microstructural changes in NDS, with the aim to demonstrate the feasibility of a new comprehensive methodological framework. Our results showed distinct imaging signatures between different NDS subtypes, with each disease condition showing characteristic (preferably GM) patterns in PET-derived glucose metabolism (SUVR), ASL-derived rCBF, and characteristic (preferably WM) patterns in DTI-derived FA. Despite subgroups of rather small sample sizes, at least an initial estimation of (diagnostic) accuracy of multimodal vs single-modal approaches could be evaluated. Nevertheless, this study cannot be competitive to large-scale single-disease/single-modality parameter studies, which is why results have to be interpreted as preliminary. Of note, the main intention of this study was to introduce a new and extendable modular framework for parameterized multimodal imaging data in NDS.

The integration of PET, ASL, and DTI allowed for distinguishing NDS from SCD and differentiating among NDS subtypes. While PET only achieved a high classification accuracy (approach A1: 92% vs. SCD, 78.1% vs. other NDS subgroups; approach A2: 80.1%; with the exception of nfvPPA where classification failed in approach A2), MRI(ASL/DTI) showed lower accuracies for specific NDS subgroups. Especially for nfvPPA and lvPPA, the multimodal approach of PET/MRI showed increased accuracies compared with PET only in both approaches A1 and A2.

Although these values are not representative, findings suggest that a combined PET/MRI framework could help to improve differential diagnostic precision. For centers without access to PET imaging, ASL and DTI-based MRI protocols may offer a viable diagnostic assistance alternative. This finding is supported by a recent work that suggested comparable spatial patterns of hypoperfusion and microstructural alterations (Bi et al., 2024).

### 4.2 Diagnosis of Alzheimer's disease

Imaging by FDG-PET remains the reference standard for detecting metabolic abnormalities in AD and other NDS subtypes (Fink et al., 2020). Its sensitivity and specificity for distinguishing AD pathology from non-AD forms of dementia have been evaluated in studies with post-mortem confirmation (Fleisher et al., 2020, Leuzy et al., 2019), further supporting its utility in clinical diagnosis. A meta-analysis demonstrated a sensitivity of 89%, specificity of 74%, and accuracy of 82% for distinguishing AD-related dementia from other dementias based on hypometabolic patterns (Fink et al., 2020). Similarly, a sensitivity of 80%, specificity of 84%, and computed accuracy of 82% were reported for AD pathology compared with other dementia pathologies (Lesman-Segev et al., 2021). However, despite the high sensitivity and diagnostic accuracy of FDG-PET, it remains a resource-intensive and costly modality that comes with radiation exposure for the patient.

### 4.3 Extension of PET by MRI

The disadvantages of PET have prompted the exploration of alternative or complementary neuroimaging techniques such as ASL and DTI, which could provide another comprehensive assessment of brain alterations in NDS. Specifically, ASL can provide valuable insights into perfusion abnormalities, which often parallel metabolic changes in NDS (Lindner et al., 2023). The current study demonstrated a high degree of regional coincidence between GM regions exhibiting hypometabolism (FDG-PET) and those with hypoperfusion (ASL), especially in AD-associated pathology, such as the inferior parietal lobule and the temporal cortex. This spatial overlap reinforces the coupling between neuronal activity, perfusion, and metabolism, which is often disrupted in NDS (Fink et al., 2020). In previous studies, ASL has been shown to detect similar spatial patterns of perfusion abnormalities as FDG-PET, though with some limitations in sensitivity due to technical variability and the inherent challenges of sequential PET/MRI acquisitions (Bi et al., 2024, Anazodo et al., 2018).

Imaging by DTI revealed significant reductions in FA in WM regions adjacent to GM areas showing hypometabolism and hypoperfusion. Hence, it seems that WM alterations may be closely tied to GM dysfunction. This is consistent with findings from recent meta-analyses showing widespread WM changes in AD, particularly involving the corpus callosum, fornix, and cingulum (Iturria-Medina and Evans, 2015, Kalva et al., 2023, Wang et al., 2020).

### 4.4 Application of support vector machine techniques

The use of SVM in this context is supported by previous work demonstrating its utility in distinguishing mild cognitive impairment and AD from healthy controls using multimodal input (Schouten et al., 2016).

Here we used three different modalities (i.e., FDG-PET, ASL, and DTI) within a single analytical framework to capture the spectrum of NDS-related changes, from metabolic dysfunction and perfusion deficits to microstructural WM damage. The results indicated that a high number of subjects could be “correctly classified” with reference to the clinical diagnosis. The high accuracy of PET in distinguishing NDS from SCD reinforces its utility in baseline disease detection. However, ASL and DTI, either combined or individually, performed comparably well in certain NDS subgroups like bvFTD and lvPPA. Notably, combining ASL and DTI provided superior accuracy for nfvPPA compared with PET only, indicating that non-invasive modalities may serve as viable diagnostic tools, especially when PET access is limited. Furthermore, the spatial overlaps seen in WBSS, where PET and ASL highlighted GM changes and DTI captured adjacent WM alterations, may help reveal the layered nature of NDS-related changes, further supporting the need for a multimodal approach to capture the full scope of disease impact across brain structures (Iturria-Medina and Evans, 2015, Kalva et al., 2023, Wang et al., 2020; Ahmadi et al., 2024). This comprehensive approach aligns with the increasing recognition that NDS cannot be fully grasped by examining single brain regions or using imaging methods in isolation (Sanz Perl et al., 2023). Instead, these diseases may be best conceptualized as network disorders, where disruptions in functional and structural connectivity across multiple brain regions could drive cognitive decline and other clinical symptoms (Zhou et al., 2020).

### 4.5 Cognitive impairment

The current findings suggest links between brain imaging results and gross cognitive impairment across NDS subtypes, as indicated by associations with MMSE scores. The MMSE is a quick and standardized tool widely used to screen for cognitive problems, assessing key cognitive domains like memory and orientation (Arevalo-Rodriguez et al., 2021, Creavin et al., 2016, Mitchell, 2017; Jia et al., 2021). However, it does not necessarily capture all aspects of cognitive decline. The MMSE also has low sensitivity for detecting changes in cognitive functioning when documenting disease courses or changes related to therapy (Ihl et al., 1992). In this study, MMSE scores correlated with measures of metabolism from PET, perfusion from ASL, and WM microstructure from DTI in AD, with correlation coefficients up to 0.69. Moreover, in lvPPA, correlations were equally pronounced, particularly with regard to metabolic markers (PET, r = 0.68–0.76). However, sensitivity and specificity of the MMSE assessment may vary in relation to various factors such as the NDS subtype or demographic factors (Spering et al., 2012).

### 4.6 Limitations and perspectives

As already stated above, sample sizes for NDS subgroups were small, which certainly limits statistical power and generalizability. The small sample sizes may also obscure subtle age-related changes in GM and WM, which are known to influence both perfusion and microstructural WM integrity (The-Editorial-Board-Neuroscience, 2020). However, the main aim of this study was to introduce a multimodal imaging-based framework for discriminating between different NDS subtypes. Furthermore, due to the retrospective character of this study, instead of healthy controls, a group of SCD (which may include also individuals with preclinical neurodegenerative changes or other neurological/psychiatric comorbidities) was used to obtain the localization of the ROIs. Thus, a future validation of the methodology of this study with age-matched cognitively normal and healthy controls should ideally be performed. Yet, entirely healthy subjects typically do not undergo PET/MRI examinations in clinical routine, which is due to its invasiveness and radiotracer-related radiation exposure. Further due to that sample size issue, a typical subdivision into training and validation datasets for machine learning was not reasonable. Future studies with extended sample sizes could be conducted with the possibility of AI-assisted applications. Another limitation refers to the correction for age-related changes in DTI and ASL. While previous research emphasized the importance of age correction in FA analyses (Behler et al., 2021), the decision in this study to correct only for observed trends rather than applying predefined correction matrices may have led to an underestimation of age-related effects. Similarly, in ASL, the small age range of the SCD group may have biased our results, since long-term age-related CBF decline has been previously documented (Dijsselhof et al., 2023).

The present analysis processed each PLD separately and subsequently averaged the resulting rCBF maps (the number of acquired volumes for each PLD were identical, i.e., 12 label/control pairs per each PLD). While this approach supports stability within the framework of this study, it does not fully exploit the potential of multi-delay acquisitions (Wang et al., 2013, Mezue et al., 2014). Model-based approaches that jointly estimate CBF and arterial transit times (ATTs) have been shown to provide more physiologically accurate perfusion estimates, particularly in samples with prolonged or heterogeneous ATT, such as older patients or patients with cerebrovascular diseases (Wang et al., 2013, van der Thiel et al., 2018, Pires Monteiro et al., 2023, Luijten et al., 2023). Multi-PLD perfusion imaging would also permit to estimate the most efficient multi-PLD acquisition regimen regarding the number of label/control pairs (Mezue et al., 2014, Woods et al., 2020). Yet, in this study, only three PLDs were considered, while ATT calculations typically use a wider spectrum of PLDs.

Regarding PET data analyses, the mean whole-brain uptake was used for an NDS-independent normalization of SUV with the restriction that, dependent on the NDS, the reference region could also include affected regions. Thus, SUVR could be improved by more sophisticated approaches with disease-specific reference regions (Borghammer et al., 2008, Nugent et al., 2020).

### 4.7 Optimization of the methodology

This study provides a framework for parameterized application of separation tools to support clinical diagnosis in NDS. In the current study, the intention was to obtain a balance of input parameters in terms of ROI localization and in terms of numbers of parameters obtained from each ROI in order to keep comparability between the smaller NDS samples as well as a comparability between the modalities (PET, DTI, ASL). Future improvements should imply the investigation of much larger samples, which would also permit the application of AI-based methods like neural networks for the differentiation of whole 3D parameter maps (Vernikouskaya et al., 2023; Dolz and Desrosiers, 2018, Volkmann et al., 2025). Furthermore, the input parameters to the SVM could be improved in the following aspects:

(i) Optimization of the localization and size of the ROIs could be obtained by analyzing an increased number of subjects for each NDS, SCD, and healthy controls.

(ii) Further ROIs, especially for AD (e.g., posterior cingulate, precuneus, and lateral (inferior) parietal lobes) could be included.

(iii) Instead of taking spherical ROIs, imaging parameters in regions defined by atlas-based parcellation masks could be used. Especially for DTI, fiber tracking masks could be used for the extraction of DTI-based parameters.

(iv) Other DTI-based parameters (e.g., mean diffusivity, axial and radial diffusivity) could be included. However, due to the dependency between these parameters, the effect of these further DTI parameters on the results of the SVM has to be investigated in future studies.

### 4.8 General methodological considerations

The SVM-based techniques aim to separate patients with a specific pathology from patients with other pathologies or from control subjects. Optimization to a sensitivity of 100% (which would be desirable, as no patient would accidentally miss a diagnosis) is usually difficult to achieve, so optimization is performed on the Youden index or on accuracy, both of which represent a good balance between sensitivity and specificity. In approach A1, optimization is based on maximum specificity in order to determine the sensitivity in the application to different NDS and thus obtain an estimate of the quality of the application. Approach A2 aims to obtain a general determination of the quality of the application to the respective pathology.

### 4.9 Conclusion

This study demonstrates the feasibility and putative clinical value of multimodal imaging techniques combining FDG-PET, ASL, and DTI, which can achieve improved diagnostic accuracy in NDS compared with single techniques. That way, this methodology may not only enhance differential diagnosis but could also provide a clinically translatable model that aligns imaging biomarkers with the pathophysiology of NDS, which could provide a solid foundation for refined diagnostic strategies and therefore improved patient care.

## Consent to participate

The requirement for written informed consent was waived due to the retrospective study design.

## Declaration of generative AI and AI-assisted technologies in the writing process

No generative AI in scientific writing was used upon submission of the paper.

## CRediT authorship contribution statement

**Joachim Strobel:** Writing – original draft, Methodology, Formal analysis, Data curation, Conceptualization. **Jan Kassubek:** Writing – review & editing, Software, Methodology. **Wolfgang Thaiss:** Writing – review & editing. **Sarah Straub-Anderl:** Resources, Data curation. **Zeljko Uzelac:** Resources, Data curation. **Sarah Jesse:** Resources, Data curation. **Laura Michelberger:** Resources, Data curation. **Christoph Solbach:** Resources, Data curation. **Ambros J. Beer:** Writing – review & editing, Supervision. **Meinrad Bee:** Writing – review & editing, Supervision. **Georg Grön:** Writing – review & editing, Resources, Formal analysis. **Hans-Peter Müller:** Writing – original draft, Visualization, Software, Methodology, Conceptualization. **Nico Sollmann:** Writing – original draft, Methodology, Conceptualization.

## Ethics approval

This monocentric study was approved by the local institutional review board (registration number 164/24). We certify that the study complies with the Declaration of Helsinki and its amendments or equivalent ethical standards.

## Funding

The authors declare that no funds, grants, or other support were received during the preparation of this manuscript.

## Declaration of competing interest

The authors declare that they have no known competing financial interests or personal relationships that could have appeared to influence the work reported in this paper.

## Data Availability

The datasets generated during and/or analyzed during the current study can be available from the corresponding author on reasonable request.

## References

[b0005] Agosta F, Basaia S, Spinelli EG, et al. Modelling pathological spread through the structural connectome in the frontotemporal dementia clinical spectrum. Brain. 2025;148:1994-2007.Nov 29 2024;doi:10.1093/brain/awae391. 10.1093/brain/awae391 PMC12129736 39611765

[b0010] Ahmadi K, Pereira JB, van Westen D, et al. Fixel-Based Analysis Reveals Tau-Related White Matter Changes in Early Stages of Alzheimer's Disease. J Neurosci. 2024;44(18)doi:10.1523/JNEUROSCI.0538-23.2024. 10.1523/JNEUROSCI.0538-23.2024 PMC11063818 38565289

[b0015] Aickin M., Gensler H. (1996). Adjusting for multiple testing when reporting research results: the Bonferroni vs Holm methods. Am. J. Public Health.

[b0020] Alsop D.C., Detre J.A., Golay X. (2015). Recommended implementation of arterial spin-labeled perfusion MRI for clinical applications: a consensus of the ISMRM perfusion study group and the European consortium for ASL in dementia. Magn. Reson. Med..

[b0025] Alsop D.C., Detre J.A., Grossman M. (2000). Assessment of cerebral blood flow in Alzheimer's disease by spin-labeled magnetic resonance imaging. Ann. Neurol..

[b0030] Anazodo U.C., Finger E., Kwan B.Y.M. (2018). Using simultaneous PET/MRI to compare the accuracy of diagnosing frontotemporal dementia by arterial spin labelling MRI and FDG-PET. Neuroimage Clin..

[b0035] Arevalo-Rodriguez I., Smailagic N., Roque-Figuls M. (2021). Mini-Mental State Examination (MMSE) for the early detection of dementia in people with mild cognitive impairment (MCI). Cochrane Database Syst. Rev..

[b0040] Behler A., Kassubek J., Muller H.P. (2021). Age-Related Alterations in DTI Metrics in the Human Brain-Consequences for Age Correction. Front. Aging Neurosci..

[b0045] Bergamino M., Keeling E., McElvogue M. (2024). White Matter Microstructure Analysis in Subjective memory complaints and Cognitive Impairment: Insights from Diffusion Kurtosis Imaging and Free-Water DTI. J. Alzheimers Dis..

[b0050] Bi S., Yan S., Chen Z. (2024). Comparison of (18)F-FDG PET and arterial spin labeling MRI in evaluating Alzheimer's disease and amnestic mild cognitive impairment using integrated PET/MR. EJNMMI Res..

[b0055] Borghammer P., Jonsdottir K.Y., Cumming P., Ostergaard K., Vang K., Ashkanian M., Vafaee M., Iversen P., Gjedde A. (2008). Normalization in PET group comparison studies–the importance of a valid reference region. Neuroimage.

[b0060] Brett M., Johnsrude I.S., Owen A.M. (2002). The problem of functional localization in the human brain. Nat. Rev. Neurosci..

[b0065] Buxton R.B., Frank L.R., Wong E.C., Siewert B., Warach S., Edelman R.R. (1998). A general kinetic model for quantitative perfusion imaging with arterial spin labeling. Magn. Reson. Med..

[b0070] Ceccarini J., Bourgeois S., Van Weehaeghe D. (2020). Direct prospective comparison of (18)F-FDG PET and arterial spin labelling MR using simultaneous PET/MR in patients referred for diagnosis of dementia. Eur. J. Nucl. Med. Mol. Imaging.

[b0075] Chen Y., Wang Y., Song Z., Fan Y., Gao T., Tang X. (2023). Abnormal white matter changes in Alzheimer's disease based on diffusion tensor imaging: a systematic review. Ageing Res. Rev..

[b0080] Chen Z., Bi S., Shan Y. (2024). Multiparametric hippocampal signatures for early diagnosis of Alzheimer's disease using (18)F-FDG PET/MRI Radiomics. CNS Neurosci. Ther..

[b0085] Cleland N.R.W., Al-Juboori S.I., Dobrinskikh E., Bruce K.D. (2021). Altered substrate metabolism in neurodegenerative disease: new insights from metabolic imaging. J. Neuroinflammation.

[b0090] Creavin S.T., Wisniewski S., Noel-Storr A.H. (2016). Mini-Mental State Examination (MMSE) for the detection of dementia in clinically unevaluated people aged 65 and over in community and primary care populations. Cochrane Database Syst. Rev..

[b0095] Dijsselhof M.B.J., Barboure M., Stritt M. (2023). The value of arterial spin labelling perfusion MRI in brain age prediction. Hum. Brain Mapp..

[b0100] Dolui S., Li Z., Nasrallah I.M., Detre J.A., Wolk D.A. (2020). Arterial spin labeling versus (18)F-FDG-PET to identify mild cognitive impairment. Neuroimage Clin..

[b0105] Dolz J., Desrosiers C. (2018). Ben AI 3D fully convolutional networks for subcortical segmentation in MRI: a large-scale study. Neuroimage.

[b0110] Du A.T., Jahng G.H., Hayasaka S. (2006). Hypoperfusion in frontotemporal dementia and Alzheimer disease by arterial spin labeling MRI. Neurology.

[b0115] Evgeniou T., Pontil M., Paliouras G., Karkaletsis V., Spyropoulos C.D. (2001). Machine Learning and Its Applications: Advanced Lectures.

[b0120] Fink H.A., Linskens E.J., Silverman P.C. (2020). Accuracy of Biomarker Testing for Neuropathologically Defined Alzheimer Disease in older adults with Dementia. Ann. Intern. Med..

[b0125] Fleisher A.S., Pontecorvo M.J., Devous M.D. (2020). Positron Emission Tomography Imaging with [18F]flortaucipir and Postmortem Assessment of Alzheimer Disease Neuropathologic changes. JAMA Neurol..

[b0130] Genovese C.R., Lazar N.A., Nichols T. (2002). Thresholding of statistical maps in functional neuroimaging using the false discovery rate. Neuroimage.

[b0135] Gorno-Tempini M.L., Hillis A.E., Weintraub S. (2011). Classification of primary progressive aphasia and its variants. Neurology.

[b0140] Grade M., Hernandez Tamames J.A., Pizzini F.B., Achten E., Golay X., Smits M. (2015). A neuroradiologist's guide to arterial spin labeling MRI in clinical practice. Neuroradiology.

[b0145] Hoffmann G., Reichert M., Göttler J., Helle M., Schmitzer L., Petzsche M.H., Zimmer C., Preibisch C., Kallmayer M., Kreiser K., Sollmann N., Liebl H., Kaczmarz S. (2025). Perfusion territory shifts in cerebrovascular diseases measured by super-selective arterial spin labeling. J. Neuroimaging.

[b0150] Holmes C.J., Hoge R., Collins L., Woods R., Toga A.W., Evans A.C. (1998). Enhancement of MR images using registration for signal averaging. J. Comput. Assist. Tomogr..

[b0155] Ihl R., Frolich L., Dierks T., Martin E.M., Maurer K. (1992). Differential validity of psychometric tests in dementia of the Alzheimer type. Psychiatry Res..

[b0160] Iturria-Medina Y., Evans A.C. (2015). On the central role of brain connectivity in neurodegenerative disease progression. Front. Aging Neurosci..

[b0165] Jack C.R., Bennett D.A., Blennow K., Carrillo M.C., Dunn B., Haeberlein S.B. (2018). NIA-AA Research Framework: Toward a biological definition of Alzheimer's disease. Alzheimers Dement..

[b0170] Jia X., Wang Z., Huang F. (2021). A comparison of the Mini-Mental State Examination (MMSE) with the Montreal Cognitive Assessment (MoCA) for mild cognitive impairment screening in chinese middle-aged and older population: a cross-sectional study. BMC Psychiatry.

[b0175] Kalva K., Zdanovskis N., Sneidere K. (2023). Whole Brain and Corpus Callosum Fractional Anisotropy differences in patients with Cognitive Impairment. Diagnostics (basel).

[b0180] Kassubek J. (2018). MRI-based neuroimaging: atypical parkinsonisms and other movement disorders. Curr. Opin. Neurol..

[b0185] Kunimatsu A., Aoki S., Masutani Y. (2004). The optimal trackability threshold of fractional anisotropy for diffusion tensor tractography of the corticospinal tract. Magn. Reson. Med. Sci..

[b0190] Le Bihan D., Breton E., Lallemand D., Grenier P., Cabanis E., Laval-Jeantet M. (1986). MR imaging of intravoxel incoherent motions: application to diffusion and perfusion in neurologic disorders. Radiology.

[b0195] Lee J., Renslo J., Wong K. (2024). Current Trends and applications of PET/MRI Hybrid Imaging in Neurodegenerative Diseases and Normal Aging. Diagnostics (basel)..

[b0200] Lesman-Segev O.H., La Joie R., Iaccarino L. (2021). Diagnostic Accuracy of Amyloid versus (18) F-Fluorodeoxyglucose Positron Emission Tomography in Autopsy-Confirmed Dementia. Ann. Neurol..

[b0205] Leuzy A., Chiotis K., Lemoine L. (2019). Tau PET imaging in neurodegenerative tauopathies-still a challenge. Mol. Psychiatry.

[b0210] Li P., Quan W., Wang Z. (2022). Early-stage differentiation between Alzheimer's disease and frontotemporal lobe degeneration: Clinical, neuropsychology, and neuroimaging features. Front. Aging Neurosci..

[b0215] Lindner T., Bolar D.S., Achten E. (2023). Current state and guidance on arterial spin labeling perfusion MRI in clinical neuroimaging. Magn. Reson. Med..

[b0220] Luijten S.P.R., Bos D., van Doormaal P.J. (2023). Cerebral blood flow quantification with multi-delay arterial spin labeling in ischemic stroke and the association with early neurological outcome. Neuroimage Clin..

[b0225] Maggipinto T., Bellotti R., Amoroso N. (2017). DTI measurements for Alzheimer's classification. Phys. Med. Biol..

[b0230] Mandelli M.L., Caverzasi E., Binney R.J. (2014). Frontal white matter tracts sustaining speech production in primary progressive aphasia. J. Neurosci..

[b0235] Meng M., Liu F., Ma Y. (2023). The identification and cognitive correlation of perfusion patterns measured with arterial spin labeling MRI in Alzheimer's disease. Alzheimers Res. Ther..

[b0240] Mezue M., Segerdahl A.R., Okell T.W., Chappell M.A., Kelly M.E., Tracey I. (2014). Optimization and reliability of multiple postlabeling delay pseudo-continuous arterial spin labeling during rest and stimulus-induced functional task activation. J. Cereb. Blood Flow Metab..

[b0245] Minoshima S., Cross D., Thientunyakit T., Foster N.L., Drzezga A. (2022). (18)F-FDG PET Imaging in Neurodegenerative Dementing Disorders: Insights into Subtype Classification, Emerging Disease Categories, and mixed Dementia with Copathologies. J. Nucl. Med..

[b0250] Mitchell A.J., Larner A.J. (2017). Cognitive Screening Instruments: A Practical Approach.

[b0255] Muddapu V.R., Dharshini S.A.P., Chakravarthy V.S., Gromiha M.M. (2020). Neurodegenerative Diseases - is Metabolic Deficiency the root Cause?. Front. Neurosci..

[b0260] Müller H.P., Kassubek J., Grön G. (2014). Impact of the control for corrupted diffusion tensor imaging data in comparisons at the group level: an application in Huntington disease. Biomed. Eng. Online.

[b0265] Müller H.P., Kassubek J. (2024). Toward diffusion tensor imaging as a biomarker in neurodegenerative diseases: technical considerations to optimize recordings and data processing. Front. Hum. Neurosci..

[b0270] Müller H.P., Turner M.R., Grosskreutz J. (2016). A large-scale multicentre cerebral diffusion tensor imaging study in amyotrophic lateral sclerosis. J. Neurol. Neurosurg. Psychiatry.

[b0275] Müller H.P., Unrath A., Ludolph A.C., Kassubek J. (2007). Preservation of diffusion tensor properties during spatial normalization by use of tensor imaging and fibre tracking on a normal brain database. Phys. Med. Biol..

[b0280] Na S., Kang D.W., Kim G.H. (2024). The Usefulness of (18)F-FDG PET to Differentiate Subtypes of Dementia: the Systematic Review and Meta-Analysis. Dement Neurocogn Disord..

[b0285] Nam G., Jeong H.J., Kang J.M. (2018). (18)F-THK5351 PET Imaging in the Behavioral Variant of Frontotemporal Dementia. Dement Neurocogn Disord..

[b0290] Nir T.M., Jahanshad N., Villalon-Reina J.E. (2013). Effectiveness of regional DTI measures in distinguishing Alzheimer's disease, MCI, and normal aging. Neuroimage Clin..

[b0295] Nugent S., Croteau E., Potvin O., Castellano C.A., Dieumegarde L., Cunnane S.C., Duchesne S. (2020). Selection of the optimal intensity normalization region for FDG-PET studies of normal aging and Alzheimer's disease. Sci. Rep..

[b0300] Okazawa H., Nogami M., Ishida S. (2024). PET/MRI multimodality imaging to evaluate changes in glymphatic system function and biomarkers of Alzheimer's disease. Sci. Rep..

[b0305] Pires Monteiro S., Pinto J., Chappell M.A. (2023). Brain perfusion imaging by multi-delay arterial spin labeling: Impact of modeling dispersion and interaction with denoising strategies and pathology. Magn. Reson. Med..

[b0310] Rascovsky K., Hodges J.R., Knopman D. (2011). Sensitivity of revised diagnostic criteria for the behavioural variant of frontotemporal dementia. Brain.

[b0315] Routier A., Habert M.O., Bertrand A. (2018). Structural, Microstructural, and Metabolic Alterations in Primary Progressive Aphasia Variants. Front. Neurol..

[b0320] Sampaio-Baptista C., Johansen-Berg H. (2017). White Matter Plasticity in the Adult Brain. Neuron.

[b0325] Sanz Perl Y., Fittipaldi S., Gonzalez Campo C. (2023). Model-based whole-brain perturbational landscape of neurodegenerative diseases. Elife.

[b0330] Scheltens P., De Strooper B., Kivipelto M. (2021). Alzheimer's disease. Lancet.

[b0335] Schouten T.M., Koini M., de Vos F., Seiler S., van der Grond J., Lechner A. (2016). Combining anatomical, diffusion, and resting state functional magnetic resonance imaging for individual classification of mild and moderate Alzheimer's disease. Neuroimage Clin..

[b0340] Schwarz C.G. (2021). Uses of Human MR and PET Imaging in Research of Neurodegenerative Brain Diseases. Neurotherapeutics.

[b0345] Sollmann N., Hoffmann G., Schramm S. (2024). Arterial Spin labeling (ASL) in Neuroradiological Diagnostics - Methodological Overview and use cases. Rofo.

[b0350] Spering C.C., Hobson V., Lucas J.A., Menon C.V., Hall J.R., O'Bryant S.E. (2012). Diagnostic accuracy of the MMSE in detecting probable and possible Alzheimer's disease in ethnically diverse highly educated individuals: an analysis of the NACC database. J. Gerontol. A Biol. Sci. Med. Sci..

[b0355] Strobel J., Yousefzadeh-Nowshahr E., Deininger K. (2024). Exploratory Tau PET/CT with [11C]PBB3 in patients with Suspected Alzheimer's Disease and Frontotemporal Lobar Degeneration: a pilot Study on Correlation with PET Imaging and Cerebrospinal Fluid Biomarkers. Biomedicines.

[b0360] Sun M., Wang Y.L., Li R., Jiang J., Zhang Y., Li W. (2022). Potential Diagnostic applications of Multi-Delay Arterial Spin labeling in Early Alzheimer's Disease: the Chinese Imaging, Biomarkers, and Lifestyle Study. Front. Neurosci..

[b0365] The-Editorial-Board-Neuroscience. Consideration of Sample Size in Neuroscience Studies. J Neurosci. 2020;40:4076-4077. doi:10.1523/JNEUROSCI.0866-20.2020. 10.1523/JNEUROSCI.0866-20.2020 PMC7244197 32434857

[b0370] Torso M., Bozzali M., Cercignani M., Jenkinson M., Chance S.A. (2020). Using diffusion tensor imaging to detect cortical changes in fronto-temporal dementia subtypes. Sci. Rep..

[b0375] Torso M., Ridgway G.R., Jenkinson M., Chance S. (2021). Frontotemporal Lobar Degeneration Neuroimaging I, the 4-repeat Tau Neuroimaging I. Intracortical diffusion tensor imaging signature of microstructural changes in frontotemporal lobar degeneration. Alzheimers Res. Ther..

[b0380] Tosun D., Schuff N., Jagust W., Weiner M.W. (2016). Alzheimer''s Disease Neuroimaging I. Discriminative Power of Arterial Spin labeling magnetic Resonance Imaging and 18F-Fluorodeoxyglucose Positron Emission Tomography changes for Amyloid-beta-positive Subjects in the Alzheimer's Disease Continuum. Neurodegener Dis.

[b0385] Tosun D., Schuff N., Rabinovici G.D. (2016). Diagnostic utility of ASL-MRI and FDG-PET in the behavioral variant of FTD and AD. Ann. Clin. Transl. Neurol..

[b0390] Unrath A., Muller H.P., Riecker A., Ludolph A.C., Sperfeld A.D., Kassubek J. (2010). Whole brain-based analysis of regional white matter tract alterations in rare motor neuron diseases by diffusion tensor imaging. Hum. Brain Mapp..

[b0395] van der Thiel M., Rodriguez C., Giannakopoulos P. (2018). Brain Perfusion Measurements using Multidelay Arterial Spin-labeling are Systematically Biased by the Number of Delays. AJNR Am. J. Neuroradiol..

[b0400] Vernikouskaya I., Müller H.P., Roselli F. (2023). AI-assisted quantication of hypothalamic atrophy in amyotrophic lateral sclerosis by convolutional neural network-based automatic segmentation. Sci. Rep..

[b0405] Volkmann H., Höglinger G.U., Grön G. (2025). MRI classification of progressive supranuclear palsy, Parkinson disease and controls using deep learning and machine learning algorithms for the identification of regions and tracts of interest as potential biomarkers. Comput. Biol. Med..

[b0410] Wang D.J.J., Alger J.R., Qiao J.X., Gunther M., Pope W.B., Saver J.L., Salamon N., Liebeskind D.S. (2013). UCLA Stroke investigators. Multi-delay multi-parametric arterial spin-labeled perfusion MRI in acute ischemic stroke - Comparison with dynamic susceptibility contrast enhanced perfusion imaging. Neuroimage Clin..

[b0415] Wang J., Zhang Y., Wolf R.L., Roc A.C., Alsop D.C., Detre J.A. (2005). Amplitude-modulated continuous arterial spin-labeling 3.0-T perfusion MR imaging with a single coil: feasibility study. Radiology.

[b0420] Wang Z., Bai L., Liu Q. (2020). Corpus callosum integrity loss predicts cognitive impairment in Leukoaraiosis. Ann. Clin. Transl. Neurol..

[b0430] Woods J.G., Chappell M.A., Okell T.W. (2020). Designing and comparing optimized pseudo-continuous Arterial Spin labeling protocols for measurement of cerebral blood flow. Neuroimage.

[b0435] Zhang N., Gordon M.L., Goldberg T.E. (2017). Cerebral blood flow measured by arterial spin labeling MRI at resting state in normal aging and Alzheimer's disease. Neurosci. Biobehav. Rev..

[b0440] Zhou J.H., Ng K.K., Liu S., Ulmer S., Jansen O. (2020). fMRI: Basics and Clinical Applications.

[b0445] Zimny A., Bladowska J., Macioszek A. (2015). Evaluation of the posterior cingulate region with FDG-PET and advanced MR techniques in patients with amnestic mild cognitive impairment: comparison of the methods. J. Alzheimers Dis..

