# Supplementary material for: Advanced neuroimaging assessment of neurodegenerative dementia syndromes: A framework for comprehensive multimodal FDG-PET, MR-perfusion, and MR-diffusion analysis
Source: Neuroimage Clin. 2026 Feb 10;49:103964. doi: 10.1016/j.nicl.2026.103964 (PMC12945645; doi:10.1016/j.nicl.2026.103964)
Supplement: Supplementary Data 1 [file mmc1.pdf]

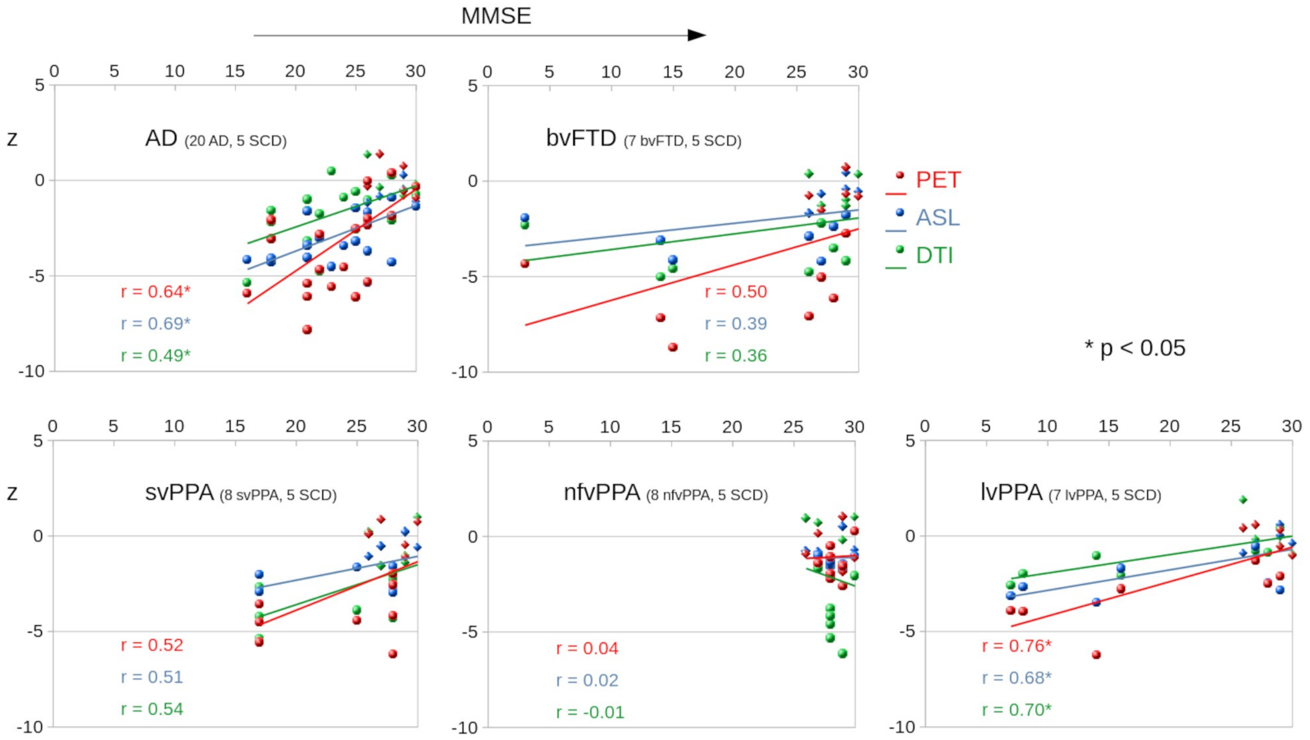

Supplementary Figure 1: Association analysis to clinical symptoms. Parameters from modality-specific ROI analysis for the respective pathology were correlated with Mini-Mental State Examination (MMSE) scores, SCD as diamonds, NDS as circles. Abbreviations: Pearson correlation value (r). Alzheimer's disease (AD), behavioral frontotemporal dementia (bvFTD), semantic variant primary progressive aphasia (svPPA), logopenic variant PPA (lvPPA), non-fluent variant PPA (nfvPPA).
