# Supplementary material for: Advanced neuroimaging assessment of neurodegenerative dementia syndromes: A framework for comprehensive multimodal FDG-PET, MR-perfusion, and MR-diffusion analysis
Source: Neuroimage Clin. 2026 Feb 10;49:103964. doi: 10.1016/j.nicl.2026.103964 (PMC12945645; doi:10.1016/j.nicl.2026.103964)
Supplement: Supplementary Data 2 [file mmc2.pdf]

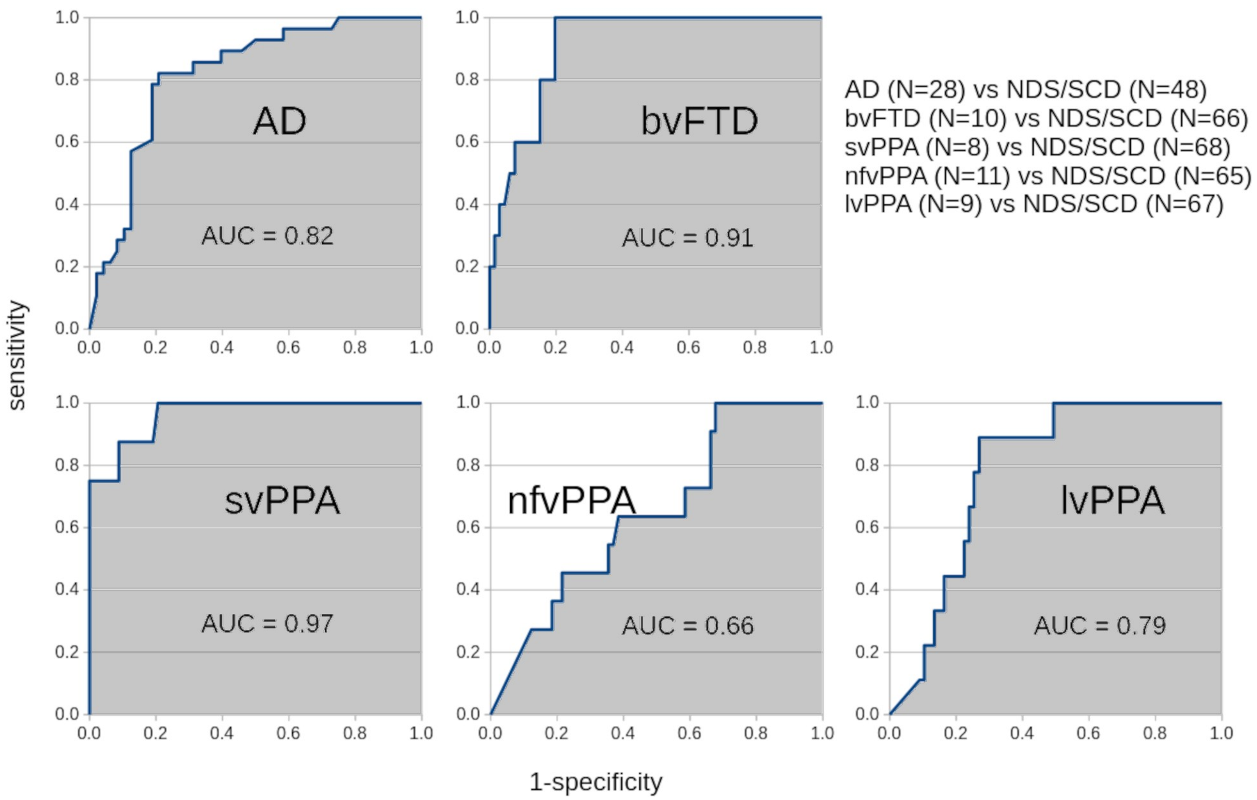

Supplementary Figure 2: Receiver operating characteristics (ROC) and area under the curve (AUC) for separating a specific neurodegenerative dementia syndrome (NDS) from other NDS and subjective cognitive decline (SCD). Abbreviations: Alzheimer's disease (AD), behavioral frontotemporal dementia (bvFTD), semantic variant primary progressive aphasia (svPPA), logopenic variant PPA (lvPPA), non-fluent variant PPA (nfvPPA).
