# Supplementary material for: Advanced neuroimaging assessment of neurodegenerative dementia syndromes: A framework for comprehensive multimodal FDG-PET, MR-perfusion, and MR-diffusion analysis
Source: Neuroimage Clin. 2026 Feb 10;49:103964. doi: 10.1016/j.nicl.2026.103964 (PMC12945645; doi:10.1016/j.nicl.2026.103964)
Supplement: Supplementary Data 3 [file mmc3.pdf]

Supplementary Table 1: *Individual classification results by PET and DTI/ASL. A subject was defined as "correctly classified" if either PET classification or DTI/ASL classification matches the initial diagnosis. Abbreviations: neurodegenerative dementia syndromes (NDS),* support vector machine (SVM), Controls (CON), *Alzheimer's disease (AD), behavioral frontotemporal dementia (bvFTD), semantic variant primary progressive aphasia (svPPA), logopenic variant PPA (lvPPA), non-fluent variant PPA (nfvPPA), diffusion tensor imaging (DTI), arterial spin labeling (ASL), positron emission tomography (PET).*

	AD	bvFTD	svPPA	nfvPPA	lgPPA	diagnosis	SVM result	alternative
subject001	0	0	0	0	0	SCD	SCD	
subject002	0	0	0	0	0	SCD	SCD	
subject003	0	0	0	0	0	SCD	SCD	
subject004	0	0	0	0	0	SCD	SCD	
subject005	0	0	0	0	0	SCD	SCD	
subject006	0	0	0	0	0	SCD	SCD	
subject007	0	0	0	0	0	SCD	SCD	
subject008	0	0	0	0	0	SCD	SCD	
subject009	0	0	0	0	0	SCD	SCD	
subject010	0	0	0	0	0	SCD	SCD	
subject011	1	0	0	1	0	AD	AD	nfvPPA
subject012	1	1	0	1	1	AD	AD	MND
subject013	0	0	1	0	0	svPPA	svPPA	
subject014	0	0	0	0	0	AD	SCD	
subject015	1	0	0	0	1	AD	AD	lgPPA
subject016	0	0	0	1	0	svPPA	svPPA	
subject017	0	0	0	0	0	AD	SCD	
subject018	1	1	1	1	0	bvFTD	bvFTD	MND
subject019	1	1	0	1	1	AD	AD	MND
subject020	0	0	0	0	1	nfvPPA	lgPPA	
subject021	1	0	0	0	1	AD	AD	lgPPA
subject022	0	1	1	0	0	svPPA	svPPA	bvFTD
subject023	1	1	0	1	0	AD	AD	MND
subject024	1	0	0	0	1	AD	AD	lgPPA
subject025	1	0	0	0	1	AD	AD	lgPPA
subject026	1	0	0	0	1	lgPPA	lgPPA	AD
subject027	1	0	0	1	0	AD	AD	
subject028	1	0	0	0	1	AD	AD	
subject029	0	0	0	0	0	nfvPPA	SCD	
subject030	1	0	0	1	1	lgPPA	lgPPA	MND
subject031	0	1	0	0	0	bvFTD	bvFTD	
subject032	1	0	0	0	0	AD	AD	
subject033	1	0	0	0	1	lgPPA	lgPPA	AD
subject034	1	0	0	0	1	lgPPA	lgPPA	AD
subject035	1	0	0	1	1	AD	AD	
subject036	0	0	0	1	0	nfvPPA	nfvPPA	
subject037	0	0	0	0	0	nfvPPA	SCD	
subject038	0	1	1	0	0	bvFTD	bvFTD	svPPA

	AD	bvFTD	svPPA	nfvPPA	lgPPA	diagnosis	SVM result	alternative
subject039	1	0	0	0	1	AD	AD	lgPPA
subject040	0	0	1	0	0	svPPA	svPPA	
subject041	0	1	0	1	0	bvFTD	bvFTD	nfvPPA
subject042	0	1	1	0	0	bvFTD	bvFTD	svPPA
subject043	0	0	0	0	0	AD	SCD	
subject044	0	1	0	1	0	bvFTD	bvFTD	nfvPPA
subject045	1	0	0	0	1	AD	AD	lgPPA
subject046	1	0	0	1	1	AD	AD	MND
subject047	1	1	0	1	1	AD	AD	MND
subject048	0	0	1	0	0	svPPA	svPPA	
subject049	0	0	0	0	0	AD	SCD	
subject050	1	0	0	1	0	AD	AD	nfvPPA
subject051	1	0	0	1	0	AD	AD	nfvPPA
subject052	0	0	1	0	0	svPPA	svPPA	
subject053	0	1	0	1	0	bvFTD	bvFTD	nfvPPA
subject054	0	0	0	0	0	lgPPA	SCD	
subject055	0	1	1	1	0	nfvPPA	nfvPPA	MND
subject056	1	0	0	0	0	AD	AD	
subject057	0	0	1	0	0	svPPA	svPPA	
subject058	0	0	0	0	1	lgPPA	lgPPA	
subject059	1	1	0	1	0	AD	AD	MND
subject060	1	1	0	1	1	AD	AD	MND
subject061	0	0	0	0	0	nfvPPA	SCD	
subject062	1	0	0	1	0	nfvPPA	nfvPPA	AD
subject063	0	0	0	1	0	nfvPPA	nfvPPA	
subject064	0	0	0	1	0	nfvPPA	nfvPPA	
subject065	0	1	0	1	1	lgPPA	lgPPA	MND
subject066	0	0	0	1	0	nfvPPA	nfvPPA	
subject067	0	1	1	1	0	bvFTD	bvFTD	MND
subject068	1	1	1	1	1	svPPA	svPPA	MND
subject069	1	0	0	0	1	lgPPA	lgPPA	AD
subject070	0	0	0	0	0	AD	SCD	
subject071	1	1	0	1	1	bvFTD	bvFTD	MND
subject072	0	1	0	0	0	bvFTD	bvFTD	
subject073	0	0	0	1	0	nfvPPA	nfvPPA	
subject074	1	1	0	1	1	AD	AD	MND
subject075	1	1	1	1	1	lgPPA	lgPPA	MND
subject076	1	1	0	1	1	AD	AD	MND
